# New Materials Based on Molecular Interaction between Hyaluronic Acid and Bovine Albumin

**DOI:** 10.3390/molecules27154956

**Published:** 2022-08-04

**Authors:** Magdalena Gadomska, Katarzyna Musiał, Piotr Bełdowski, Alina Sionkowska

**Affiliations:** 1 Department of Biomaterials and Cosmetic Chemistry, Faculty of Chemistry, Nicolaus Copernicus University in Toruń, Gagarin 7, 87-100 Toruń, Poland; 2 Institute of Mathematics and Physics, Faculty of Chemical Technology and Engineering, Bydgoszcz University of Technology J.J. Śniadeckich, 85-796 Bydgoszcz, Poland

**Keywords:** molecular interaction, hyaluronic acid, bovine albumin, biomaterials

## Abstract

In this work, the interactions between hyaluronic acid and bovine serum albumin were investigated. The film-forming properties of the mixture were proven, and the mechanical and surface properties of the films were measured. The results showed the interactions between hyaluronic acid and albumin, mainly by hydrogen bonds. Molecular docking was used for the visualization of the interactions. The films obtained from the mixture of hyaluronic acid possessed different properties to films obtained from the single component. The addition of bovine serum albumin to hyaluronic acid led to a decrease in the mechanical properties, and to an increase in the surface roughness of the film. The new materials that have been obtained by blending can form a new group of materials for biomedicine and cosmetology.

## 1. Introduction

Synthetic and natural polymers are composed of repeating fragments called mers [1]. Synthetic polymers, due to their properties, have been used in many industries. They are widely used in cosmetic preparations as film-forming substances in nail varnishes, mascaras, and hair styling cosmetics. Natural polymers, which are produced by living organisms, are an alternative to synthetic polymers. This is because biopolymers are non-toxic, biocompatible, and biodegradable, which makes them almost ideal raw materials in various industries [2,3,4]. Special attention has been paid to polysaccharides because they are the most abundant carbohydrates found in nature. Polysaccharides and their modified forms have found wide application in the medical and pharmaceutical industries—for example, as drug carriers [5]. Selected polysaccharides may show anti-inflammatory, antiviral, and antibacterial properties, making them suitable raw materials for producing biomedical materials. The most common natural polysaccharides are cellulose, starch, chitosan, and hyaluronic acid [6]. One of the widely used polysaccharides in the cosmetic and biomedical fields is hyaluronic acid (HA). HA is a water-soluble compound whose structure includes D-glucuronic acid and N-acetyl-D-glycosamine [7]. HA can easily form hydrogen bonds due to the presence of many hydroxyl groups and, in this way, has the ability to bind water [8]. Hyaluronic acid can be found in rooster crests, the vitreous of the eye, the human umbilical cord, human synovial fluid, and the skin. The content of hyaluronic acid varies depending on the tissue. Low-molecular-weight HA is involved in wound healing, ovulation, and embryonic development, while high-molecular-weight HA has an anti-inflammatory effect [9]. Hyaluronic acid also exhibits diverse biological functions in living organisms by interacting with a large number of proteins and cell surface receptors.

The properties of HA can be modified by blending with other polymers and/or biopolymers. In recent years, blends of hyaluronic acid with other polymers have been studied for new material development [10]. New materials may show improved properties that are important in biomedical applications—for example, in scaffold fabrication. Polymer scaffolds for biomedical applications can be composed of blends of hyaluronic acid with synthetic polymers and/or another natural polymer. Proteins, such as albumins, can also be considered as a component of the blend with polysaccharides [2]. The most common types of albumins in the biomaterials industry are human serum albumin (HSA), bovine serum albumin (BSA), and egg albumin [11]. Serum albumin is the most common protein that can be found in the blood of vertebrate organisms, and it is made of as many as 585 amino acids, of which 35 are cysteine, thanks to which it is possible to create disulfide bridges. Its function is to transport substances in the plasma. BSA is used as a model protein in in vitro studies. BSA is characterized by very good stability, low toxicity, and low production costs. Until now, albumin has been used to treat burns. Moreover, albumin-based materials have been used to treat liver cirrhosis, pancreatic cancer, lung cancer, and metastatic breast cancer. Protein biomaterials are characterized by the ease of delivering active substances into the bloodstream. Moreover, albumin is completely non-toxic, biodegradable, and biocompatible. In the human body, it is broken down into smaller peptides or amino acids under the influence of proteinases, which are also non-toxic and do not elicit a response by the immune system [12,13,14,15,16].

Biopolymer blend preparation by dissolution in the same solvent allows for avoiding protein denaturation. The specific interaction between the blend components is often called miscibility. The most common interactions in the blends are hydrogen bonding, ionic and dipole, and charge-transfer complexes. The interactions between HA and BSA can determine the properties of the blend. Moreover, the interactions between hyaluronic acid and albumin can be important from a biomedical point of view because the mixture of hyaluronic acid and albumin can mimic the synovial fluid, and may influence the tribological properties of cartilage [12,17,18]. However, it should be emphasized that biopolymers and biopolymer blends dedicated to biomaterial fabrication must meet several basic conditions. They must be non-toxic, biocompatible, and non-carcinogenic. They cannot react with a living organism, causing unwanted reactions [19,20]. The fabrication of new biomaterials based on mixtures of biopolymers is a new trend in materials sciences; nevertheless, within the last three decades, an increasing interest in new materials based on blends of two or more polymers has been observed [21,22,23,24,25,26,27]. The potential applications of biopolymer blends in the biomedical field can be broad and may include drug delivery systems, tissue engineering, wound healing, or gene therapy [28]. It was shown that polymer blend films could be effectively used as wound dressings because they absorb wound exudate while maintaining a sufficiently moist environment that promotes healing processes. Unfortunately, proteins are very susceptible to degradation processes, so modification is required to obtain materials more resistant to degradation. Modifying the structure of polysaccharides by protein addition may lead to new materials for tissue engineering and drug delivery systems. With the growing needs of the medical market, new biomaterials need to be created that have better mechanical properties and cause fewer side effects, and, at the same time, are characterized by better therapeutic effectiveness [8,9,29,30,31].

In this work, polymer thin films composed of pure hyaluronic acid of three different molecular weights and their mixtures with BSA have been researched. The interactions between two macromolecules have been studied experimentally and molecular docking has been used for visualization of these interactions. The mechanical and physicochemical properties of the obtained polymeric films were investigated and compared. To the best of our knowledge, the interactions between HA with different molecular weight and BSA have not been studied yet.

## 2. Results

### 2.1. FTIR

**Figure 1:**
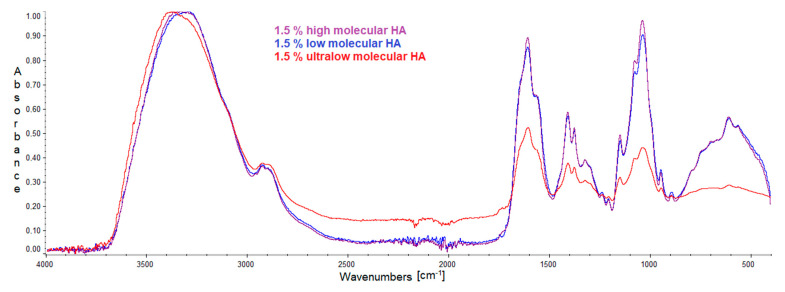
IR spectrum of high-, low-, and ultralow molecular weight hyaluronic acid.

**Figure 2:**
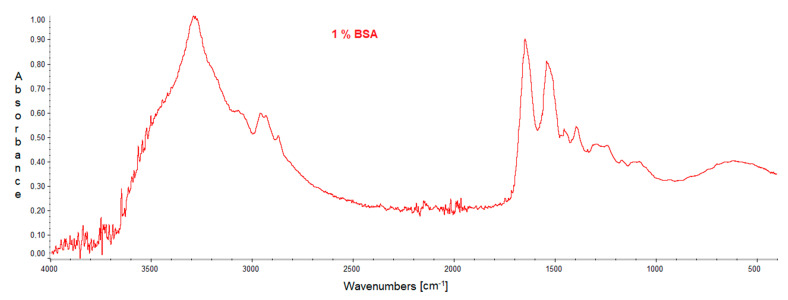
IR spectrum of the film obtained from 1% solution of BSA.

**Figure 3:**
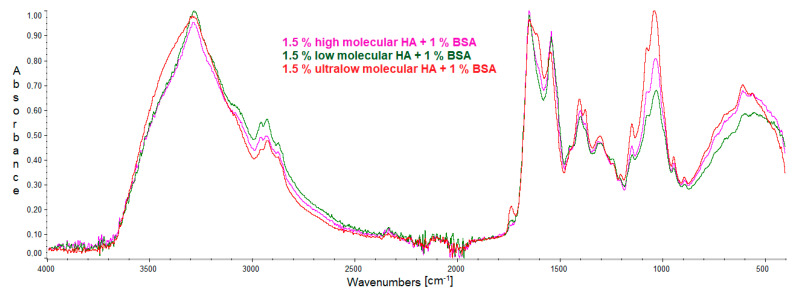
IR spectrum of the film obtained from the mixtures of high-, low-, and ultralow-molecular-weight HA with 1% solution of BSA.

IR spectra were recorded to determine the interaction between hyaluronic acid and albumin. Individual spectra of pure compounds and mixtures are presented in the figures below (Figure 1, Figure 2 and Figure 3). The positions of the bands are presented in the tables below (Table 1, Table 2 and Table 3).

The IR spectra for the three hyaluronic acids are almost identical, regardless of their molecular weight. Minor differences in the wavenumber may be caused by a slight change in conditions when performing spectrometric measurements. The most remarkable difference can be seen between the high- and low-molecular-weight HA spectra and the ultralow-molecular HA spectrum. This may be due to the significant difference in molecular weight and, therefore, the peaks in the ultralow-molecular HA spectrum are much smaller. The obtained IR spectra confirm the presence of amide, hydroxyl, and carboxyl groups in the tested compounds.

There are characteristic N-H and C-N bonds in albumin, the presence of which was confirmed in the IR spectrum. The wavenumbers corresponding to these bonds are, respectively, 1650 cm^−1^ and 1541 cm^−1^. In the IR spectra of proteins, there are Amide A, Amide I, and Amide II bands. The Amide A band in BSA is located at 3290 cm^−1^, whereas Amide I and II are located at 1650 cm^−1^ and 1541 cm^−1^, respectively.

There are no significant differences between the spectra of HA–bovine serum albumin mixtures. One can see the difference between the spectra of pure albumin and the mixture with hyaluronic acid. The spectrum of albumin does not show an intense peak corresponding to approximately 590 cm^−1^. There was a decrease in the peaks corresponding to O-H and C-H bonds and an increase in the COC binding peak. The spectra of HA–bovine albumin mixtures show a peak corresponding to COH binding, which is not present in albumin. Moreover, a shift in the band in the region of OH/Amide A after the mixing of the two biopolymers has been observed. There is also a difference in the position of the bands at 1407 cm^−1^ in HA; after the addition of BSA, these bands have been shifted to lower wavenumbers. The abovementioned changes in the IR spectra indicate an interaction between albumin and hyaluronic acid. The interaction occurs mainly via hydrogen bonds.

### 2.2. Mechanical Properties

The mechanical properties of membranes made of pure hyaluronic acid of three different molecular weights and membranes made by mixing hyaluronic acid with bovine serum albumin have been measured. The obtained results are presented in the tables below (Table 4 and Table 5). No films were obtained from the mixture of ultralow-molecular-weight HA with BSA; therefore, their mechanical properties were not investigated. HA with ultralow molecular weight cannot form a film that is possible to remove from the plastic plate and prepare the sample for the mechanical testing. It can be concluded that ultralow-molecular-weight HA has very weak film-forming properties.

The mechanical properties of the obtained polymeric films vary depending on the molecular weight of the hyaluronic acid. The highest value of Young’s modulus (E_mod_) was obtained for the high-molecular-weight HA. The highest value of elongation at break was obtained for the low-molecular-weight HA.

The addition of bovine serum albumin to the hyaluronic acid solution significantly affects the mechanical properties of the obtained polymer films. The tested films made of mixtures of HA and albumin had lower Young’s modulus, tensile strength, and elongation at break values.

Changes in the mechanical properties following the addition of albumin may suggest interactions between hyaluronic acid and albumin. This has been confirmed in spectrometric tests with FTIR. The weaker mechanical properties after the addition of bovine serum albumin may suggest that the hydrogen bonds between HA and albumin are weaker than the hydrogen bonds between HA molecules.

### 2.3. AFM

**Figure 4:**
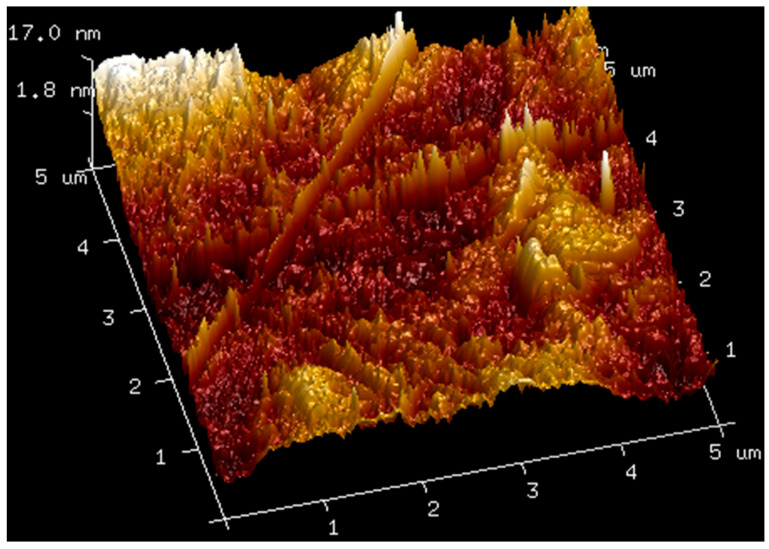
AFM visualization of the surface of film obtained from the solution of 1.5% high-molecular HA.

**Figure 5:**
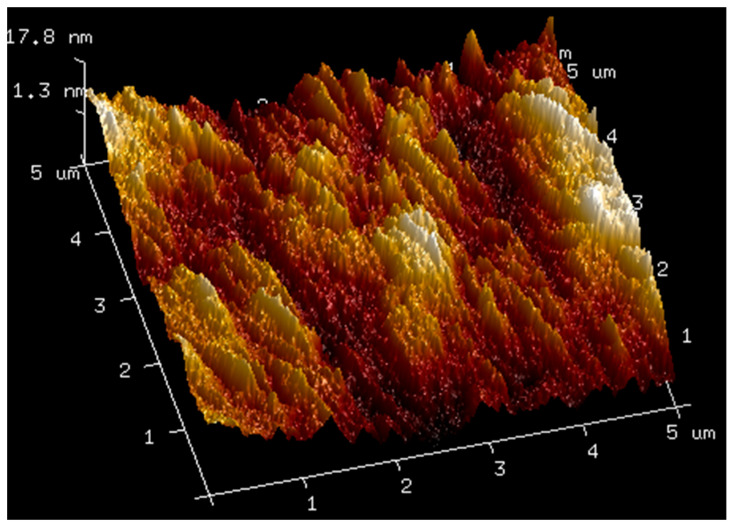
AFM visualization of the surface of film obtained from the solution of 1.5% low-molecular HA.

**Figure 6:**
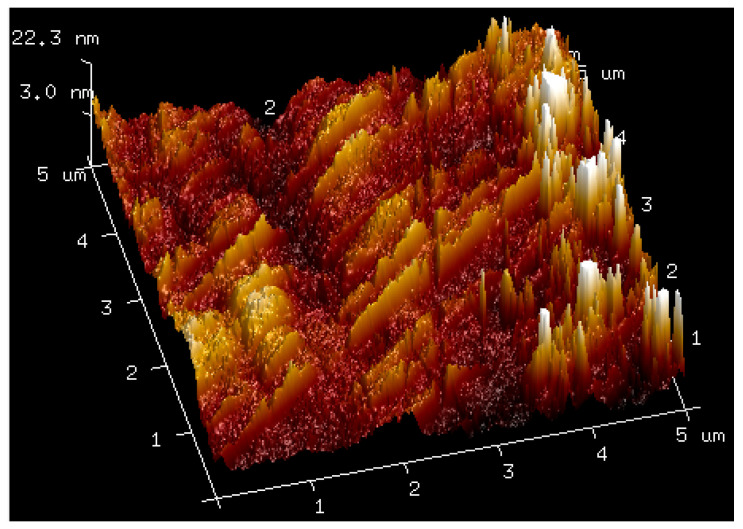
AFM visualization of the surface of film obtained from the solution of 1.5% ultralow-molecular HA.

**Figure 7:**
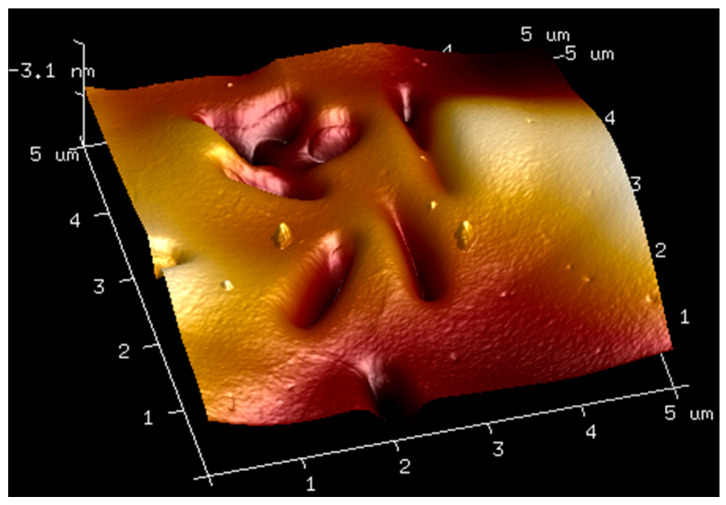
AFM visualization of the surface of film obtained from the solution of 1.5% high-molecular HA with 1% BSA.

**Figure 8:**
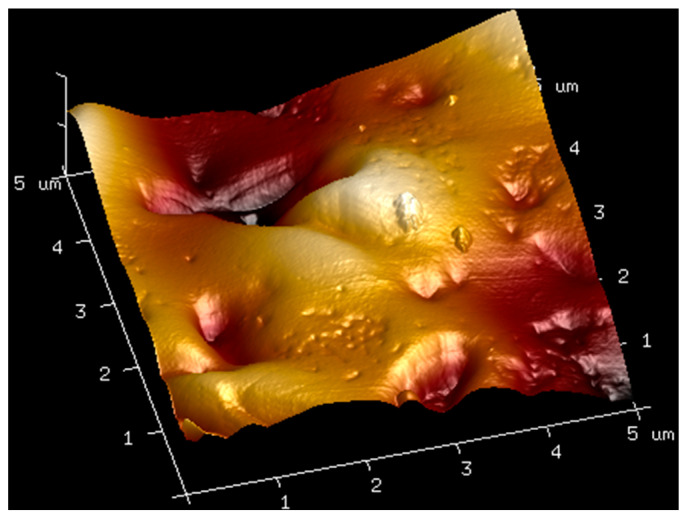
AFM visualization of the surface of film obtained from the solution of 1.5% low-molecular HA with 1% BSA.

AFM analysis of membranes composed of pure hyaluronic acid of three different molecular weights and of membranes made by mixing hyaluronic acid with bovine serum albumin was carried out. The obtained results are shown in the figures below (Figure 4, Figure 5, Figure 6, Figure 7 and Figure 8). The Ra values (arithmetic mean of the absolute values of surface height deviations measured from the plane) and the Rq values (mean square of the height deviations) are listed in Table 6.

Rq values are always higher than Ra values. In the case of the films prepared from pure hyaluronic acid, there are no significant differences in their surface roughness. The addition of BSA to hyaluronic acid causes tremendous changes in the surface roughness of the films. The surface of the blends is rougher than the surface of the films obtained from pure HA. This fact could indicate interactions between the components of the mixtures.

### 2.4. Molecular Docking

**Figure 9:**
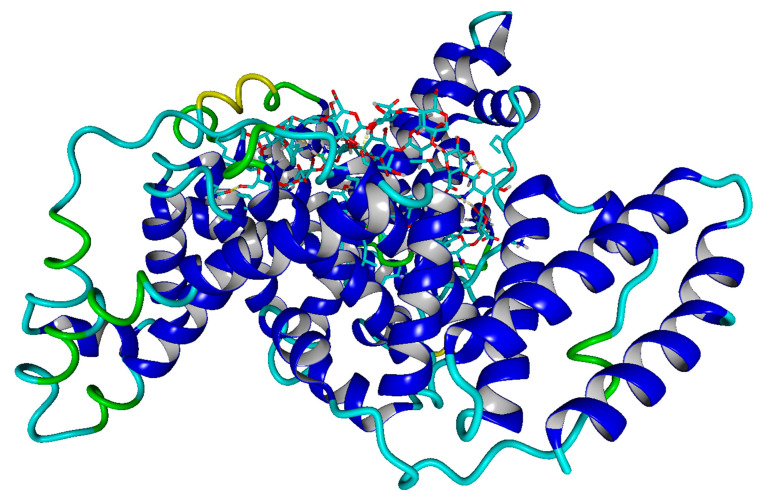
Visualization of BSA–HA complex obtained from docking. BSA is represented in ribbon-like structure, whereas HA is shown as stick model.

**Figure 10:**
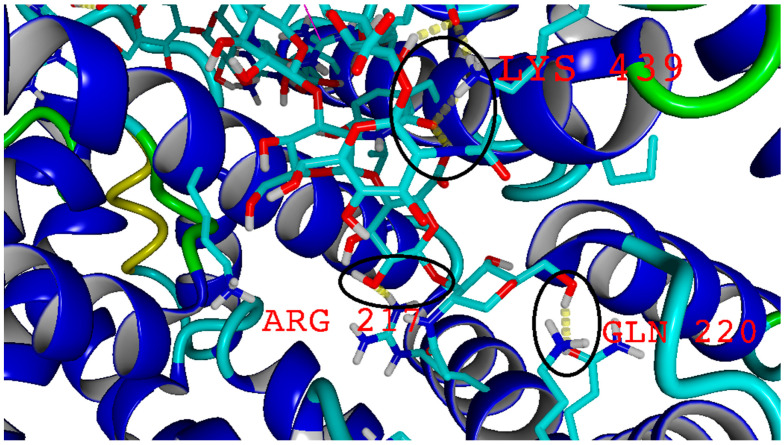
Visualization of hydrogen bonds (yellow dotted lines) inside the BSA–HA complex. Black circles and amino acids forming hydrogen bonds have been added for better visualization.

Visualization of BSA–HA complex obtained from molecular docking and visualization of hydrogen bonds have been shown in Figure 9 and Figure 10, respectively. 

The results of the molecular docking experiment show that HA tends to bind in subdomain IB, with some overlap with other domains, mainly IIIA and IIIB (Table S1 in Supplementary Information). HA binds mainly with the hydroxyl group to charged amino acids such as glutamic acid, lysine, or arginine. Additionally, it can create ionic interactions between carboxyl groups in HA and ARG or LYS side chains.

## 3. Discussion

The physicochemical properties of hyaluronic acid depend on its molecular weight. This fact was confirmed by the conducted spectrometric measurements, mechanical tests, and AFM visualization. Moreover, its ability to create films made of the blend of bovine albumin and HA also depends on the molecular weight of HA. In general, bovine serum albumin can be combined with hyaluronic acid, and thin films can be obtained. However, the addition of albumin reduces the mechanical properties of the hyaluronic acid films. This may be due to the interaction of albumin and hyaluronic acid and the formation of weaker bonds between BSA and HA than those between HA molecules. The spectrometric studies performed by IR confirmed the interactions between the hyaluronic acid molecule and albumin. Nevertheless, the shifts in the bands in the IR spectra were not too large. However, even a small shift in the IR bands may suggest interactions due to the formation of hydrogen bonds between the components of the mixtures. In fact, in both macromolecules, there are several places that can be involved in hydrogen bond formation.

Despite the slight differences in the roughness of the surfaces of the three hyaluronic acid films, diametrically different values were obtained for the HA–BSA mixtures. This phenomenon is connected with the existence of interactions based on hydrogen bonding in the mixtures. By modifying hyaluronic acid with BSA, we can obtain a material with a different roughness that will better adhere to a specific surface. Thus, blending HA and BSA can lead to better membranes, medical dressings, and cosmetic masks. The obtained polymer films based on hyaluronic acid and their mixtures with albumin can be used in the biomaterials and cosmetics industries, e.g., as biomimetic coatings and adhesives. The blends of hyaluronic acid and albumin can also be used in drug delivery systems. However, more research is needed to investigate the biological activity, biocompatibility, or rheological properties of the materials obtained.

Although blends of HA and BSA have not been widely studied, it should be emphasized that HA–protein interactions and new materials based on the blends have been studied already. For example polymer–polymer interactions, such as hydrophobic and electrostatic interactions, have been studied in a hyaluronic acid and collagen mixture, and new matrices based on the blends have been obtained by Taguchi et al. [32,33]. It was also found that collagen–hyaluronic acid membranes for applications in regenerative medicine can be obtained by self-assembly [34]. Moreover, porous hybrid scaffolds based on collagen and hyaluronic acid have been prepared by Lee et al. [35]. Based on hyaluronic acid–tyrosine and human-like collagen, multifunctionalized hydrogels have been fabricated by Liu et al. [36]. As the extracellular matrix can be mimicked by biopolymer blends in several forms, we can expect that, in the future, not only membranes and thin films made of the blend of HA and BSA will be fabricated. HA modified by BSA may form, for example, injectable hydrogels, which can play an important role in soft-tissue filling and repair. An injectable hydrogel composed of hyaluronic acid and other proteins has been designed to mimic the extracellular matrix for vascular cell growth and wound closure by Ying et al. [37].

As the preparation of a hyaluronic acid blend with other biopolymers is neither a closed nor a completed topic, we can expect several research works concerning this aspect. Several new materials for both biomedical and cosmetic applications can be proposed, not only based on HA and BSA, but also based on other blends. In the scientific literature, one can find reports about blends of HA with the following polymers: carboxymethyl cellulose [38], pullulan [39], silk fibroin [40], alginate [41], chondroitin sulfate [42], phospholipids [43], and many other polymers and biopolymers [10].

## 4. Materials and Methods

### 4.1. Materials

Hyaluronic acid (HA) of high molecular weight, HA of low molecular weight, and HA of ultralow molecular weight were purchased from the cosmetic company zrobsobiekrem.pl (Prochowice, Poland). Probumin^®^ Bovine Serum Albumin (BSA) was purchased from Merck (Merck Life Science, Poznań, Poland). The molecular weight of HA was proven by viscometry techniques and was as follows:

M_HA high molecular_ = 8.434 × 10^5^ g/mol

M_HA low molecular_ = 6.327 × 10^5^ g/mol

M_HA ultralow molecular_ = 3.445 × 10^4^ g/mol

### 4.2. Preparation of Solutions

Solutions of pure biopolymers were prepared by dissolution in water with the following concentrations: BSA with 1% high-molecular-weight HA, with 1.5% low-molecular-weight HA, and with 1.5% ultralow-molecular-weight HA.

### 4.3. Preparation of Polymer Mixtures and Films

Each of the three hyaluronic acid solutions was mixed with a 1% BSA solution. To do this, 15 g of albumin solution was added to 15 g of HA solution, followed by stirring for 15 min on a magnetic stirrer. Then, 25 g of the obtained mixtures and pure solutions of the two types of HA were poured onto plastic plates (dimension 10 cm × 10 cm). It took a week for the solvent to evaporate and form the polymer film.

### 4.4. FTIR Spectroscopy

Infrared spectra were recorded on a Nicolet iS10 FTIR spectrophotometer equipped with a diamond crystal ATR device (Thermo Fisher Scientific, Waltham, MA, USA). The IR spectra were registered for membranes obtained from hyaluronic acid and its mixture with albumin. Spectra were recorded with a resolution of 4 cm^−1^ with 64 scans and scored in the range of 400–4000 cm^−1^. The spectra were processed using Omnic Spectra 2009.

### 4.5. Mechanical Properties

From the obtained polymer films, shapes with the following dimensions were cut: width 1 cm and length 5 cm. Mechanical properties such as Young’s modulus [GPa], tensile strength [MPa], and elongation at break [%] were tested on a Zwick & Roell Z.0.5 testing machine. (Zwick & Roell, Ulm, Germany). Seven to 10 trials were performed for each film. The initial parameters of the test program were as follows: preload 0.1 MPa, test speed 50 mm/min, preload speed 5 mm/min. Data were collected in testXpert II 2017. Statistical analysis was performed using the Q-Dixon test in MS Excel.

### 4.6. AFM

The surface structure of the polymer films was analyzed based on atomic force microscope images obtained with the MultiMode Scanning Probe Microscope Nanoscale IIIa (Digital Instruments Veeco Metrology Group, Santa Barbara, CA, USA) operating in trigger mode, in an air atmosphere at room temperature. The roughness parameters were calculated from the 5 µm × 5 µm scan area using the Nanoscope software (Bruker Optoc GmbH, Ettlingen, Germany).

### 4.7. Molecular Docking

To obtain molecular details on HA deposition at BSA (PDB code: 3 v 03), we performed molecular docking of BSA–HA complexes using the VINA method [44], with default parameters and point charges initially assigned according to the AMBER14 force field [45] (the HA molecule of the molar mass of 8 kDa was parametrized by applying the GLYCAM06 force field) and then damped to mimic the less polar Gasteiger charges used to optimize the AutoDock scoring function. The setup was achieved with the YASARA molecular modeling program [46,47]. The best hit of 50 runs with −10 kcal/mol free energy of binding was selected as distinctive complexes varying with the position of HA. We obtained 32 complexes by varying the HA position as shown in Table S1. Additionally, we performed a hydrogen bond analysis similar to one in [18].

## 5. Conclusions

Hyaluronic acid can be mixed efficiently with BSA, but the film-forming properties depend on the molecular weight of HA. The interactions between the components of the blend are mainly achieved by hydrogen bonds. The molecular docking experiment shows the most likely binding sites, as well as the fact that hydrogen bonds form mainly with charged amino acids and hydroxyl groups in HA. The addition of bovine albumin causes a deterioration in the mechanical properties of hyaluronic acid. The surface roughness is greater in the blends than in hyaluronic acid films. The obtained biomaterials can be used in the medical and cosmetic industries as film-forming substances and biomimetic adhesives. Moreover, the HA–BSA blends can find potential applications in tissue engineering and regeneration, as injectable hydrogels and as components of cosmetic formulations. The conventional clinical protocol with HA can be modified by the proper addition of BSA.

## Figures and Tables

**Table 1:** The positions of bands in IR spectra (wavenumbers) for high-, low-, and ultralow-molecular-weight hyaluronic acid.

Function Group	Vibrations	Bond Position for 1.5% High-Molecular HA [cm^−1^]	Band Position for 1.5% Low-Molecular HA [cm^−1^]	Band Position for 1.5% Ultralow-Molecular HA [cm^−1^]
**O-H**	Stretching	3332	3291	3366
**C-H**	Stretching	2928	2927	2921
**N-H**	Deformative	1607	1607	1605
**C-N**	Stretching	1409	1409	1407
**COH**	Stretching	1038	1036	1036
**COC**	Stretching	609	608	607

**Table 2:** The positions of bands in IR spectra (wavenumbers) for bovine serum albumin.

Function Group	Vibrations	Band Position for 1% BSA [cm^−1^]
O-H/Amide A	Stretching	3290
C-H	Stretching	2959
N-H	Deformative	1650
C-N	Stretching	1541
O-H	Stretching	1395
COC	Stretching	622

**Table 3:** The positions of bands in IR spectra (wavenumbers) for the mixtures of high-, low-, and ultralow-molecular-weight HA with bovine serum albumin.

Function Group	Vibrations	Band Position for 1.5% High-Molecular HA with 1% BSA [cm^−1^]	Band Position for 1.5% Low-Molecular HA with 1% BSA [cm^−1^]	Band Position for 1.5% Ultralow-Molecular HA with 1% BSA [cm^−1^]
O-H/Amide A	Stretching	3286	3285	3292
C-H	Stretching	2933	2928	2929
N-H	Deformative	1650	1650	1651
C-N	Stretching	1542	1542	1548
O-H	Deformative	1402	1399	1405
COH	Stretching	1034	1031	1041
COC	Stretching	607	554	608

**Table 4:** Mechanical properties of the film made of the high-, low-, and ultralow-molecular-weight hyaluronic acid.

Sample	Young’s Modulus E_mod_ [GPa]	Tensile Strength [MPa]	Elongation at Break [%]
1.5% high-molecular HA	0.733 ± 0.463	58.53 ± 6.24	9.57 ± 5.0
1.5% low-molecular HA	0.21 ± 0.45	58.37 ± 1.19	16.13 ± 6.2
1.5% ultralow-molecular HA	0.621 ± 0.351	48.07 ± 12.0	2.71 ± 0.5

**Table 5:** Mechanical properties of films made from a mixture of hyaluronic acid solution with bovine serum albumin.

Sample	Young’s Modulus E_mod_ [GPa]	Tensile Strength [MPa]	Elongation at Break [%]
1.5% high-molecular HA with 1% BSA	0.58 ± 0.331	54.18 ± 4.65	0.17 ± 0.9
1.5% low-molecular HA with 1% BSA	0.55 ± 0.379	53.6 ± 5.86	4.33 ± 1.1

**Table 6:** Ra and Rq values for films obtained from high, low, and ultralow HA and mixtures of HA with BSA.

Sample	Ra [nm]	Rq [nm]
1.5% high-molecular-weight HA	3.12	3.99
1.5% low-molecular-weight HA	3.81	4.78
1.5% ultralow-molecular-weight HA	3.87	5.21
1.5% high-molecular-weight HA with 1% BSA	36.8	45.7
1.5% low-molecular-weight HA with 1% BSA	51.8	65.1

## Data Availability

Not applicable.

## Supplementary Materials

The following supporting information can be downloaded at: https://www.mdpi.com/article/10.3390/molecules27154956/s1, Figure S1: The distribution of amino acids in BSA contributes to a total number of Hydrogen bonds with HA. Table S1: Results of molecular docking of HA on BSA. Values in the third column represent the name of the amino acid and its number in the primary structure.

## Sample Availability

Samples of the compounds of hyaluronic acid and bovine serum albumin are available from the authors.
