# Supplementary material for: New Materials Based on Molecular Interaction between Hyaluronic Acid and Bovine Albumin
Source: Molecules. 2022 Aug 4;27(15):4956. doi: 10.3390/molecules27154956 (PMC9370313; doi:10.3390/molecules27154956)
Supplement: Supplementary file 1 [file molecules-27-04956-s001.zip › molecules-1825781-supplementary.pdf]

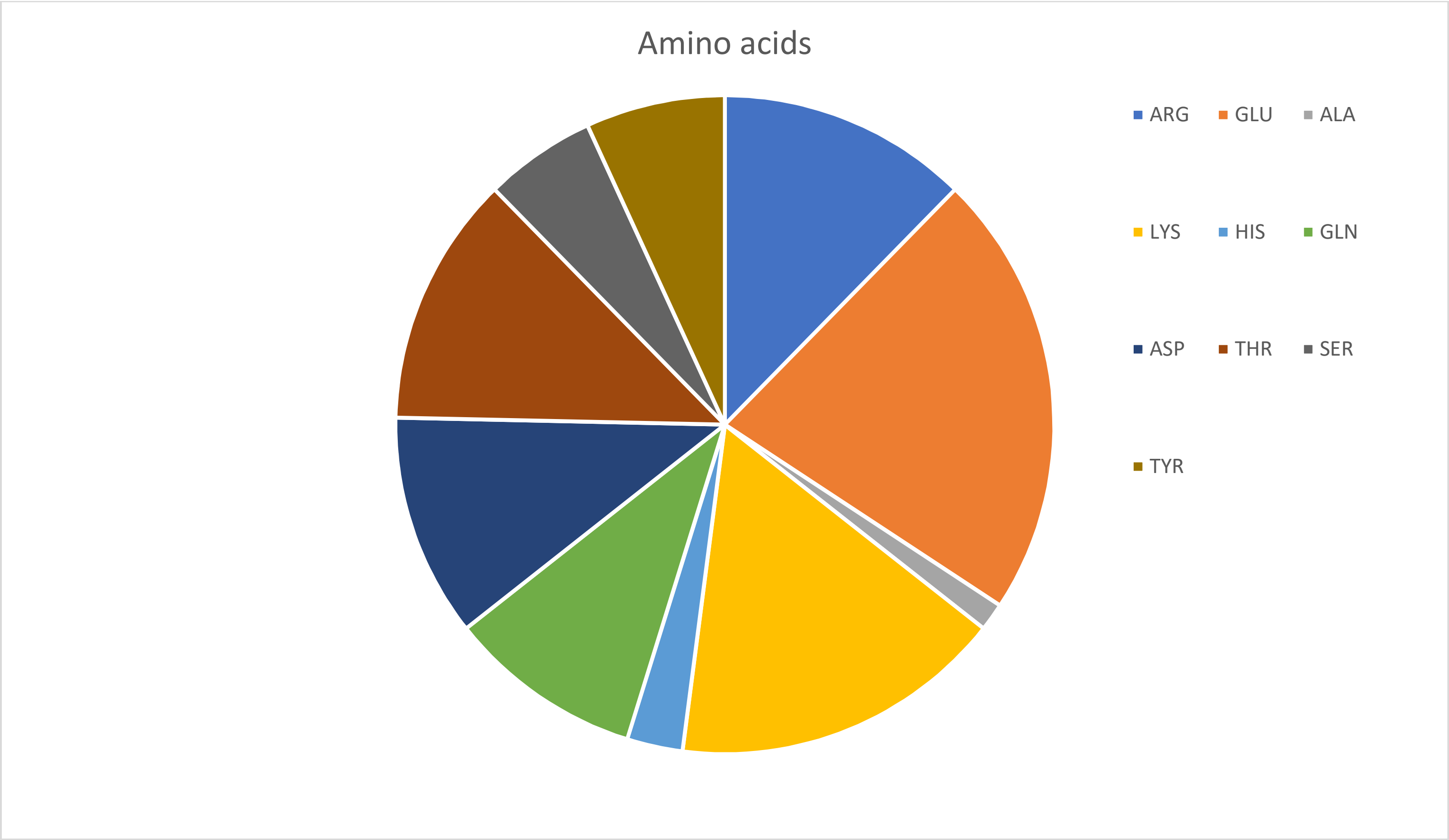

Figure S1. The distribution of amino acids in BSA contributes to a total number of Hydrogen bonds with HA.

Table S1. Results of molecular docking of HA on BSA. Values in the third column represent the name of the amino acid and its number in the primary structure.

Cluster	Bind energy[kcal/mol]	Contacting receptor residues
1	8,16	SER 109 PRO 110 ASP 111 LEU 112 LYS 114 LEU 115 LYS 116 PRO 117 ARG 144 HIS 145 PRO 179 GLU 182 THR 183 ARG 185 GLU 186 LYS 187 LEU 189 THR 190 ALA 193 ARG 194 ARG 217 GLN 220 LYS 221 GLU 291 VAL 292 GLU 293 LYS 294 PRO 338 GLU 339 TYR 340 ALA 341 VAL 342 GLU 399 PRO 420 GLU 424 ARG 427 SER 428 LYS 431 THR 434 ARG 435 CYS 436 THR 438 LYS 439 GLU 443 PRO 446 CYS 447 ASP 450 TYR 451 LEU 454 ILE 455 ARG 458 PRO 516 THR 518 GLU 519 ILE 522
2	8,139	ASP 108 SER 109 PRO 110 ASP 111 LEU 112 LYS 114 LEU 115 ARG 144 HIS 145 TYR 155 TYR 156 LYS 159 VAL 163 PRO 179 LYS 180 GLU 182 THR 183 MET 184 ARG 185 GLU 186 LYS 187 VAL 188 LEU 189 THR 190 ALA 193 ARG 194 ARG 217 GLN 220 HIS 287 GLU 291 VAL 292 GLU 293 LYS 294 GLU 339 TYR 340 ALA 341 VAL 342 GLU 399 GLU 424 SER 428 LYS 431 THR 434 ARG 435 LYS 439 GLU 443 PRO 446 CYS 447 ASP 450 TYR 451 LEU 454 ILE 455 ARG 458 THR 518 GLU 519 ILE 522
3	8,116	ASP 108 SER 109 ASP 111 LYS 114 ARG 144 HIS 145 GLU 182 THR 183 ARG 185 GLU 186 LYS 187 LEU 189 THR 190 SER 192 ALA 193 ARG 194 ARG 217 GLN 220 LYS 273 GLU 291 VAL 292 GLU 293 LYS 294 ILE 297 PRO 338 GLU 339 TYR 340 ALA 341 VAL 342 GLU 399 PRO 420 VAL 423 GLU 424 ARG 427 SER 428 LYS 431 THR 434 ARG 435 LYS 439 GLU 443 PRO 446 CYS 447 ASP 450 TYR 451 LEU 454 ILE 455 ARG 458 PRO 516 THR 518 GLU 519 ILE 522
4	7,606	ASP 108 SER 109 PRO 110 ASP 111 LYS 114 LEU 115 LYS 116 PRO 117 ARG 144 HIS 145 PRO 179 GLU 182 THR 183 ARG 185 GLU 186 LYS 187 LEU 189 THR 190 ALA 193 ARG 194 ARG 217

		GLU 291 VAL 292 GLU 293 LYS 294 ASP 295 ALA 296 ILE 297 PRO 298 GLU 299 ASN 300 LEU 301 PRO 302 PRO 303 LEU 304 ARG 336 HIS 337 PRO 338 GLU 339 PRO 420 GLU 424 ARG 427 SER 428 LYS 431 THR 434 ARG 435 LYS 439 GLU 443 PRO 446 CYS 447 ASP 450 TYR 451 LEU 454 ILE 455 ARG 458 THR 518 GLU 519 ILE 522
5	7,568	ASP 107 SER 109 PRO 110 ASP 111 LYS 114 LEU 115 LYS 116 PRO 117 ARG 144 HIS 145 LEU 178 GLU 182 ARG 185 GLU 186 LEU 189 ALA 193 GLU 399 GLN 403 GLN 416 VAL 417 SER 418 THR 419 PRO 420 THR 421 GLU 424 ARG 427 SER 428 LYS 431 THR 434 ILE 455 ARG 458 LEU 462 LYS 465 THR 466 VAL 468 LYS 499 LYS 504 LEU 505 PRO 516 ASP 517 THR 518 GLU 519 GLN 521 ILE 522 LYS 523 THR 526 GLU 530 LYS 533
6	7,554	ASP 108 SER 109 PRO 110 ASP 111 LEU 112 PRO 113 LYS 114 LEU 115 LYS 116 PRO 117 ARG 144 HIS 145 PRO 146 TYR 147 LEU 178 ILE 181 GLU 182 THR 183 ARG 185 GLU 186 LYS 187 LEU 189 THR 190 SER 192 ALA 193 ARG 194 ARG 196 ARG 217 GLN 220 LYS 273 GLU 291 GLU 293 LYS 294 ASP 295 ALA 296 ILE 297 PRO 338 GLU 339 ALA 341 VAL 342 GLU 399 GLU 424 ARG 427 SER 428 LYS 431 THR 434 ARG 435 LYS 439 SER 442 GLU 443 PRO 446 CYS 447 ASP 450 TYR 451 ARG 458 PRO 516 ASP 517 THR 518 GLU 519 ILE 522
7	7,542	ASP 108 ASP 111 LEU 112 PRO 113 LYS 114 LEU 115 LYS 116 PRO 117 ARG 144 HIS 145 TYR 155 LYS 159 LEU 178 GLU 182 THR 183 ARG 185 GLU 186 LYS 187 LEU 189 THR 190 SER 192 ALA 193 ARG 194 ARG 217 GLN 220 GLU 284 HIS 287 CYS 288 GLU 291 VAL 292 GLU 293 LYS 294 PRO 338 GLU 339 ALA 341 VAL 342 GLU 424 ARG 427 SER 428 LYS 431 THR 434 ARG 435 THR 438 LYS 439 PRO 440 GLU 443 CYS 447 ASP 450 TYR 451 ILE 455 ARG 458 PRO 516 ASP 517 THR 518 GLU 519 ILE 522
8	7,478	GLN 32 GLN 33 THR 83 TYR 84 GLY 85 MET 87 HIS 105 ASP 107 ASP 108 SER 109 PRO 110 ASP 111 LEU 112 PRO 113 LYS 114 LEU 115 LYS 116 PRO 117 ARG 144 HIS 145 LEU 178 PRO 179 GLU 182 THR 183 ARG 185 LEU 189 SER 192 ALA 193 GLU 399 GLN 403 PRO 420 GLU 424 ARG 427 SER 428 LYS 431 THR 434 ARG 435 ARG 458 LEU 462 LYS 465 PRO 516 ASP 517 THR 518 GLU 519 GLN 521 ILE 522
9	7,4	TYR 155 TYR 156 ASN 158 LYS 159 GLY 162 VAL 163 GLU 166 LYS 180 THR 183 MET 184 GLU 186 LYS 187 THR 190 ARG 194 ARG 217 GLN 220 GLU 276 LYS 280 PRO 281 GLU 284 HIS 287 CYS 288 GLU 291 VAL 292 GLU 293 LYS 294 GLU 339 TYR 340 ALA 341 VAL 342 ARG 435 LYS 439 PRO 440 SER 442 GLU 443 MET 445 PRO 446 CYS 447 ASP 450 TYR 451
10	7,39	GLN 33 CYS 34 PRO 35 PHE 36 ASP 37 THR 83 ASP 107 SER 109 PRO 110 ASP 111 LEU 112 PRO 113 LYS 114 LEU 115 GLU 125 LYS 132 LYS 136 TYR 139 GLU 140 ARG 143 ARG 144 LEU 178 PRO 179 GLU 182 THR 183 ARG 185 GLU 186 LYS 187 LEU 189 THR 190 ARG 194 ARG 217

		GLN 220 LYS 294 PRO 338 GLU 339 TYR 340 ALA 341 VAL 342 LEU 345 GLU 399 PRO 420 THR 421 GLU 424 LYS 431 THR 434 ARG 435 THR 438 LYS 439 GLU 443 PRO 446 CYS 447 ASP 450 TYR 451 LEU 462 LYS 465 PRO 516 THR 518 GLU 519 ILE 522
11	7,359	ASP 111 LEU 112 PRO 113 LYS 114 LEU 115 LYS 116 PRO 117 ARG 144 HIS 145 TYR 155 TYR 156 LYS 159 GLY 162 VAL 163 LEU 178 PRO 179 LYS 180 GLU 182 THR 183 MET 184 ARG 185 GLU 186 LYS 187 LEU 189 THR 190 ARG 194 ARG 217 GLN 220 GLU 276 LYS 280 GLU 284 HIS 287 CYS 288 GLU 291 VAL 292 GLU 293 LYS 294 TYR 340 ALA 341 VAL 342 GLU 399 LYS 431 THR 434 ARG 435 LYS 439 PRO 446 CYS 447 ASP 450 TYR 451 PRO 516 ASP 517 THR 518 GLN 521
12	7,334	LYS 114 LEU 115 LYS 116 PRO 117 TYR 155 TYR 156 LYS 159 VAL 163 GLU 166 PRO 179 LYS 180 GLU 182 THR 183 MET 184 ARG 185 GLU 186 LYS 187 VAL 188 LEU 189 THR 190 ARG 194 ARG 217 LYS 221 HIS 287 CYS 288 GLU 291 VAL 292 GLU 293 LYS 294 PRO 338 GLU 339 ALA 341 GLU 399 LYS 431 THR 434 ARG 435 LYS 439 GLU 443 PRO 446 CYS 447 ASP 450 TYR 451
13	7,297	GLN 33 ALA 78 SER 79 ARG 81 GLU 82 TYR 84 GLY 85 ASP 86 MET 87 ASP 89 GLU 92 HIS 105 ASP 107 SER 109 PRO 110 ASP 111 LEU 112 LYS 114 LEU 115 LYS 116 PRO 117 ARG 144 HIS 145 GLU 182 ARG 185 GLU 186 LEU 189 THR 190 SER 192 ALA 193 GLU 399 TYR 400 GLN 403 PRO 420 THR 421 VAL 423 GLU 424 ARG 427 SER 428 LYS 431 ARG 435 TYR 451 ILE 455 ARG 458 LEU 462 LYS 465 PRO 516 THR 518 GLU 519 GLN 521 ILE 522
14	7,275	SER 109 ASP 111 LYS 114 LEU 115 LYS 116 PRO 117 ILE 141 ARG 144 HIS 145 LEU 178 GLU 182 THR 183 ARG 185 GLU 186 LYS 187 LEU 189 THR 190 ARG 194 ARG 217 GLU 291 VAL 292 GLU 293 LYS 294 GLU 399 TYR 400 GLN 403 GLU 424 ARG 427 LYS 431 THR 434 ARG 435 THR 438 TYR 451 PRO 516 ASP 517 THR 518 GLU 519 GLN 521 ILE 522
15	7,244	GLN 32 GLN 33 THR 83 TYR 84 GLY 85 MET 87 SER 104 HIS 105 ASP 107 SER 109 PRO 110 ASP 111 LYS 114 LEU 115 LYS 116 PRO 117 ARG 144 HIS 145 LEU 178 GLU 182 ARG 185 GLU 186 LEU 189 SER 192 ALA 193 GLU 399 TYR 400 GLN 403 SER 418 THR 419 PRO 420 THR 421 VAL 423 GLU 424 ARG 427 SER 428 LYS 431 THR 434 ARG 435 ARG 458 LEU 462 LYS 465 THR 466 PRO 516 ASP 517 THR 518 GLU 519 GLN 521 ILE 522 THR 526 GLU 530
16	7,244	SER 109 PRO 110 ASP 111 PRO 113 LYS 114 LEU 115 LYS 116 PRO 117 LEU 178 PRO 179 GLU 182 THR 183 ARG 185 GLU 186 LYS 187 LEU 189 THR 190 ARG 194 ARG 217 GLN 220 LYS 221 GLU 291 LYS 294 TYR 340 ALA 341 VAL 342 GLU 399 TYR 400 GLN 403 PRO 420 VAL 423 GLU 424 ARG 427 LYS 431 THR 434 ARG 435 LYS 439 GLU 443 PRO 446 CYS 447 ASP 450 TYR 451 THR 514 LEU 515 PRO 516 ASP 517 THR 518 GLU 519 GLN 521 ILE 522

17	7,193	SER 109 PRO 110 ASP 111 LEU 112 PRO 113 LYS 114 LEU 115 PRO 117 ARG 144 HIS 145 LEU 178 PRO 179 GLU 182 THR 183 ARG 185 GLU 186 LEU 189 THR 190 SER 192 ALA 193 GLU 399 GLN 403 PRO 415 GLN 416 SER 418 THR 419 PRO 420 THR 421 GLU 424 ARG 427 SER 428 LYS 431 THR 434 ARG 435 ILE 455 ARG 458 LEU 462 LYS 465 THR 466 VAL 468 TYR 496 VAL 497 PRO 498 LYS 499 ALA 500 ASP 517 THR 518 GLU 519 GLN 521 ILE 522 GLU 530 LYS 533
18	7,182	ASP 108 SER 109 ASP 111 LEU 112 LYS 114 LEU 115 LYS 116 PRO 117 ARG 144 HIS 145 LEU 178 PRO 179 GLU 182 THR 183 ARG 185 GLU 186 LEU 189 THR 190 SER 192 ALA 193 PHE 394 GLU 399 TYR 400 GLN 403 GLU 424 ARG 427 SER 428 LYS 431 THR 434 ARG 435 LEU 454 ILE 455 ARG 458 PRO 516 ASP 517 THR 518 GLU 519 GLN 521 ILE 522
19	7,177	SER 109 ASP 111 LYS 114 LEU 178 PRO 179 GLU 182 THR 183 ARG 185 GLU 186 LYS 187 LEU 189 THR 190 ARG 194 ARG 217 GLN 220 LYS 294 PRO 338 GLU 339 TYR 340 ALA 341 VAL 342 GLU 399 PRO 415 GLN 416 VAL 417 SER 418 THR 419 PRO 420 VAL 423 GLU 424 ARG 427 LYS 431 THR 434 ARG 435 THR 438 LYS 439 GLU 443 PRO 446 CYS 447 ASP 450 TYR 451 LYS 465 THR 466 VAL 468 TYR 496 VAL 497 LYS 499 LEU 505 ASP 511 PRO 516 ASP 517 THR 518 GLU 519 ILE 522 LYS 523 THR 526 GLU 530 LYS 533
20	7,162	ASP 108 SER 109 PRO 110 ASP 111 LYS 114 LEU 115 LYS 116 PRO 117 ARG 144 HIS 145 TYR 155 TYR 156 LYS 159 LEU 178 GLU 182 THR 183 MET 184 ARG 185 GLU 186 LYS 187 LEU 189 THR 190 ARG 194 ARG 217 GLN 220 GLU 276 GLU 284 HIS 287 CYS 288 GLU 291 VAL 292 GLU 293 LYS 294 PRO 338 GLU 339 TYR 340 ALA 341 VAL 342 GLU 399 GLU 424 ARG 427 LYS 431 THR 434 ARG 435 LYS 439 GLU 443 PRO 446 CYS 447 ASP 450 TYR 451 ARG 458 LEU 462 PRO 516 ASP 517 THR 518 GLU 519 ILE 522
21	7,159	TYR 84 GLU 100 LEU 103 SER 104 HIS 105 ASP 107 ASP 108 SER 109 PRO 110 ASP 111 LYS 114 LEU 115 LYS 116 PRO 117 ARG 144 HIS 145 TYR 147 LEU 178 GLU 182 ARG 185 LEU 189 ARG 196 ILE 202 GLN 203 LYS 204 PHE 205 GLY 206 GLU 207 ARG 208 LYS 211 VAL 228 THR 231 LYS 232 VAL 234 THR 235 THR 238 HIS 246 ASP 323 ALA 324 SER 418 PRO 420 THR 421 GLU 424 ARG 427 LYS 431 VAL 461 LEU 462 GLU 464 LYS 465 THR 466 PRO 467 PRO 516 THR 518 GLU 519 ILE 522
22	7,156	ASP 108 LYS 114 LEU 115 LYS 116 PRO 117 ARG 144 HIS 145 LEU 178 GLU 182 ARG 185 GLU 186 LEU 189 THR 190 SER 192 ALA 193 ARG 196 LYS 396 LEU 397 GLY 398 GLU 399 TYR 400 GLY 401 GLN 403 ASN 404 GLU 424 ARG 427 SER 428 LYS 431 THR 434 ARG 435 TYR 451 LEU 454 ILE 455 ARG 458 PRO 516 ASP 517 THR 518 LYS 520 GLN 521 LYS 524 LYS 544 MET 547 GLU 548 VAL 551 ASP 555

23	7,132	SER 109 PRO 110 ASP 111 LYS 114 LEU 115 LYS 116 PRO 117 HIS 145 LEU 178 PRO 179 GLU 182 THR 183 ARG 185 GLU 186 LEU 189 THR 190 SER 192 ALA 193 GLU 399 TYR 400 GLN 403 PRO 415 GLN 416 VAL 417 SER 418 THR 419 PRO 420 THR 421 GLU 424 ARG 427 SER 428 LYS 431 ARG 435 TYR 451 ILE 455 ARG 458 LEU 462 LYS 465 THR 466 VAL 468 TYR 496 VAL 497 LYS 499 ALA 500 PRO 516 ASP 517 THR 518 GLU 519 GLN 521 ILE 522 LYS 533
24	7,124	LEU 115 LYS 116 PRO 117 TYR 155 TYR 156 LYS 159 LEU 178 PRO 179 LYS 180 GLU 182 THR 183 MET 184 ARG 185 GLU 186 LYS 187 LEU 189 THR 190 ARG 194 ARG 217 LYS 221 GLU 284 HIS 287 CYS 288 GLU 291 VAL 292 GLU 293 LYS 294 PRO 338 GLU 339 ALA 341 LYS 431 THR 434 ARG 435 LYS 439 PRO 440 GLU 443 PRO 446 CYS 447 ASP 450 TYR 451 THR 518
25	7,035	ASP 108 ASP 111 LYS 114 LEU 115 LYS 116 PRO 117 PRO 119 HIS 145 LEU 178 PRO 179 GLU 182 THR 183 ARG 185 GLU 186 LYS 187 LEU 189 THR 190 SER 192 ALA 193 ARG 194 ARG 196 LEU 197 ARG 217 GLN 220 CYS 288 GLU 291 VAL 292 GLU 293 LYS 294 PRO 338 GLU 339 TYR 340 ALA 341 GLU 424 ARG 427 SER 428 LYS 431 THR 434 ARG 435 LYS 439 GLU 443 PRO 446 CYS 447 ASP 450 TYR 451 LEU 454 ILE 455 ASN 457 ARG 458 VAL 461 PRO 516 ASP 517 THR 518 GLU 519 ILE 522
26	7,031	ASP 108 LYS 116 PRO 117 HIS 145 LEU 178 GLU 182 THR 183 ARG 185 GLU 186 LYS 187 LEU 189 THR 190 SER 192 ALA 193 ARG 196 LEU 197 ARG 217 LYS 221 GLU 291 VAL 292 GLU 293 LYS 294 PRO 338 GLU 339 ALA 341 GLU 424 ARG 427 SER 428 LYS 431 THR 434 ARG 435 LYS 439 GLU 443 PRO 446 CYS 447 ASP 450 TYR 451 LEU 454 ILE 455 ASN 457 ARG 458 VAL 461 PRO 516 ASP 517 THR 518 ILE 522
27	7,014	LYS 114 LEU 115 LYS 116 PRO 117 HIS 145 GLU 182 THR 183 ARG 185 GLU 186 LYS 187 LEU 189 THR 190 SER 192 ALA 193 ARG 194 ARG 217 GLU 291 GLU 293 LYS 294 ILE 297 LEU 304 ARG 336 HIS 337 PRO 338 GLU 339 TYR 340 ALA 341 LYS 377 GLU 399 TYR 400 GLN 403 GLU 424 ARG 427 SER 428 LYS 431 THR 434 ARG 435 SER 442 GLU 443 PRO 446 CYS 447 TYR 451 LEU 454 ILE 455 ARG 458 PRO 516 ASP 517 THR 518 GLU 519 GLN 521 ILE 522
28	6,998	SER 109 PRO 110 ASP 111 LEU 112 PRO 113 LYS 114 LEU 115 ARG 144 TYR 155 GLU 182 THR 183 ARG 185 GLU 186 LYS 187 LEU 189 THR 190 ARG 194 ARG 217 LYS 273 GLU 276 LYS 280 GLU 284 HIS 287 CYS 288 GLU 291 VAL 292 GLU 293 LYS 294 PRO 338 GLU 339 ALA 341 VAL 342 GLU 399 TYR 400 GLN 403 GLU 424 ARG 427 LYS 431 THR 434 ARG 435 THR 438 LYS 439 GLU 443 ASP 450 TYR 451 ARG 458 ASP 517 THR 518 GLN 521 ILE 522
29	6,939	SER 109 PRO 110 ASP 111 LYS 114 LEU 115 LYS 116 PRO 117 ARG 144 HIS 145 GLU 182 ARG 185 GLU 186 LEU 189 LEU 397 GLY 398 GLU 399 TYR 400 GLY 401 GLN 403 ASN 404 PRO 420

		VAL 423 GLU 424 ARG 427 LYS 431 LYS 465 PRO 516 ASP 517 THR 518 GLU 519 LYS 520 GLN 521 ILE 522 LYS 524 LYS 544 MET 547 GLU 548 VAL 551 ASP 555
30	6,936	ASP 108 SER 109 ASP 111 LYS 114 LYS 116 PRO 117 HIS 145 PRO 179 GLU 182 THR 183 ARG 185 GLU 186 LYS 187 LEU 189 THR 190 SER 192 ALA 193 ARG 194 ARG 217 GLU 291 GLU 293 LYS 294 GLU 339 ALA 341 VAL 342 GLU 399 GLN 403 GLU 424 ARG 427 SER 428 LYS 431 THR 434 ARG 435 THR 438 LYS 439 PRO 440 GLU 443 PRO 446 CYS 447 ASP 450 TYR 451 ARG 458 LEU 462 PRO 516 ASP 517 THR 518 GLU 519 GLN 521 ILE 522
31	6,931	ASP 108 SER 109 PRO 110 ASP 111 LYS 114 LEU 115 LYS 116 PRO 117 ARG 144 HIS 145 LEU 178 PRO 179 GLU 182 THR 183 ARG 185 GLU 186 LEU 189 GLU 399 TYR 400 GLN 403 SER 418 PRO 420 VAL 423 GLU 424 ARG 427 SER 428 LYS 431 THR 434 ARG 435 THR 438 ARG 458 LEU 462 LYS 465 THR 466 PRO 516 ASP 517 THR 518 GLU 519 GLN 521 ILE 522
32	6,898	ASP 111 LYS 114 LEU 115 LYS 116 PRO 117 ILE 141 ARG 144 HIS 145 LEU 178 PRO 179 GLU 182 THR 183 ARG 185 GLU 186 LYS 187 LEU 189 SER 192 ALA 193 GLU 399 TYR 400 GLN 403 GLU 424 ARG 427 SER 428 LYS 431 THR 434 ARG 435 THR 438 LYS 439 ILE 455 ARG 458 LEU 515 PRO 516 ASP 517 THR 518 LYS 520 GLN 521 LYS 524 VAL 551 ASP 555
